# Protein Bread Fortification with Cumin and Caraway Seeds and By-Product Flour

**DOI:** 10.3390/foods7030028

**Published:** 2018-02-25

**Authors:** Bouchra Sayed Ahmad, Thierry Talou, Evita Straumite, Martins Sabovics, Zanda Kruma, Zeinab Saad, Akram Hijazi, Othmane Merah

**Affiliations:** 1 Laboratoire de Chimie Agro-industrielle (LCA), Université de Toulouse, INRA, 31030 Toulouse, France; bouchra.sayed.ahmad@hotmail.com (B.S.A.); thierry.talou@ensiacet.fr (T.T.); 2 Research Platform of Environmental Science, Doctoral School of Science and Technology, Lebanese University, Campus Rafic Hariri, BP 5 Hadath-Beirut, Lebanon; zsaad2002@yahoo.com (Z.S.); hijazi_akram@hotmail.com (A.H.); 3 Department of Food Technology, Faculty of Food Technology, Latvia University of Agriculture, Rigas iela 22, Jelgava LV-3001, Latvia; evita.straumite@llu.lv (E.S.); martins.sabovics@llu.lv (M.S.); zanda.kruma@llu.lv (Z.K.); 4 Département Génie Biologique, Université Paul Sabatier, IUT A, 24 rue d’Embaquès, 32000 Auch, France

**Keywords:** caraway, cumin, bread quality, by-products, radical scavenging activity, total phenolic

## Abstract

Malnutrition continues to be a key health problem in developing regions. The valorization of food waste appears as an ideal way to prevent malnutrition and improve people’s access to food. Cumin (*Cuminum cyminum* L.) and caraway (*Carum carvi* L.) oilseeds are commonly used for cuisine and medicinal purposes. However, remaining cakes after oil extraction are usually underutilized. In order to assess the usefulness of these by-products in food applications, this study investigated the effect of their addition to protein bread formulations. Different levels (2, 4 and 6%) of whole seeds and cakes flour were used in the study. Fortified protein bread samples were compared to control protein bread and evaluated for their sensory, color, moisture, hardness properties, nutritional values as well as their biological activity. Results indicated that bread fortification shows a significant effect on bread properties depending on fortification level. A higher acceptability was observed specially for bread fortified with by-products flour. Increased tendencies of color darkness, moisture content, bread hardness, nutritional values as well as total phenolic content and radical scavenging activity compared to control bread were observed as the percentage of fortification increased in both cases. The overall results showed that the addition of cumin and caraway seeds and by-product flour can improve the antioxidant potential and overall quality of protein bread.

## 1. Introduction

Wheat bread is a very popular foodstuff in the daily diets of most of the population with more than 32 million tons of annual consumption in the European market only. With the increasing awareness of consumption of healthy food, production of bread from whole wheat flour is highly recommended in bakery industries. Whole wheat flour led to improvement of the nutritional values and fiber content of the final bread, while the aesthetic value and the sensory properties are negatively affected by comparison with bread made from white flour [1]. In this context, vital wheat protein appears as an adequate additive which can enhance not only the texture and the shelf life of the bread, but also a bread enriched in protein is obtained [2].

Cumin (*Cuminum cyminum* L.) and caraway (*Carum carvi* L.) belong to the Apiaceae family. Originated from the Mediterranean region and India, they are widely cultivated in temperate regions and used as spices in many popular cuisines [3]. For centuries, cumin and caraway seeds have been grown for food and medicinal uses owing to their high nutritional values with presence of high content of proteins, fiber, minerals, bioactive compounds, volatile and vegetable oils [4]. Nevertheless, vegetable oils extracted from cumin and caraway seeds are considered as a rich source of petroselinic acid (C18:1*n*-12) which is a rare monounsaturated fatty acid used as a raw material in chemical and cosmetic industries. Petroselinic acid is a precursor of both lauric and adipic acids which are used for the production of detergents and surfactants and the synthesis of nylon polymer, respectively. Petroselinic acid is also an important ingredient used in skin hydrations and anti-aging formulas [5]. However, after oil extraction, the remaining cakes from cumin and caraway seeds, so-called by-products, are underutilized and generally considered as waste. Recently, there is a growing focus on valorization of seed by-products for their potential health benefits as antioxidant and antimicrobial agents due to their richness in bioactive compounds [5].

Consumers increasingly request functional foods, taking into account their higher content in nutraceutical compounds and their direct contribution in preventing nutrition-related diseases [6]. Therefore, supplementing bread with nutritious additives in order to boost its physical and nutritional properties is very trendy nowadays [7]. Previous studies have focused on bread fortification with different kinds of plant seed and by-products such as pumpkin seed [8], grape seed [9], fennel seed [10], and by-products of walnut kernel and brown linseed [11,12,13]. In spite of having different health benefits, cumin and caraway seeds and by-products have not yet attracted much attention. Due to the fact that they could be regarded as functional agents to improve bread quality, the aim of this study is to investigate the effect of the addition of cumin and caraway powder seeds and by-products on the sensory, textural and biological properties of protein enriched bread. Obtained bread is dedicated to people who are on a high-protein diet due to the use of high content of wheat protein.

## 2. Materials and Methods

### 2.1. Seed Extraction

Extrusion was done by a Single-screw (Model OMEGA 20, Dana Brevini, Villeurbanne, France) press with the following parameters: a motor (0.75  kW, 230  V of maximal tension, 5.1 A of maximal intensity), a screw length of 18  cm, a pitch screw of 1.8  cm, with an internal diameter of 1.4  cm, a channel depth of 0.5  cm, and a sleeve of 2.5  cm of internal diameter equipped with a filter-pierced outlet for liquid at the end of the screw and at the surface of the nozzles. The filter section was 2 mm in diameter to separate extracted oil. The feed rate and the screw rotation speed were maintained constant to 15 g∙min^−1^ (0.9 kg∙h^−1^) and 40 rpm, respectively. The nozzle diameter used in the pressing of cumin and caraway seed was 5 mm. The nozzle/screw distance was 3 cm. The screw press was first run for 15 min without seed material but with heating via an electrical resistance-heating ring attached around the press barrel, to raise the screw press barrel temperature to the desired value. Cumin and caraway obtained as by-products by extrusion process were used for further research. 

### 2.2. Raw Materials for Protein Bread Preparation 

Whole wheat flour (GmbH Rigas Dzirnavnieks, Riga, Latvia), wheat protein isolate Arise 5000 (MGP Ingredients, Athinsone, Kansas, USA), sugar (GmbH Nordic Sugar, Riga, Latvia), salt, dry yeast (GmbH S.I. Lesaffre, Marcq-en-Baroeul, France) were procured from the local market of Jelgava, Latvia; while cumin and caraway seeds were purchased from the local market of Toulouse, France. 

### 2.3. Protein Bread Making Technology 

**Figure 1:**
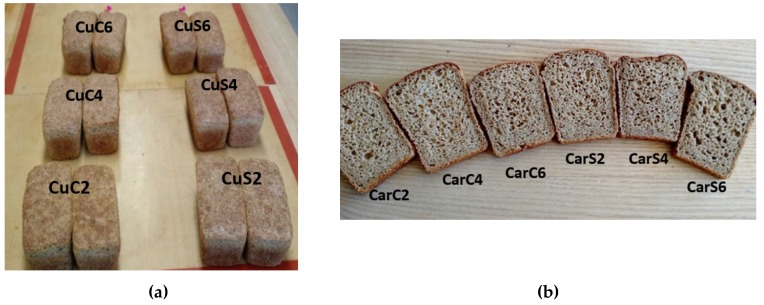
Bread fortified with cumin (**a**) and caraway (**b**) seeds and by-products.CuC2: 2% of cumin cake; CuC4:4% of cumin cake; CuC6: 6% of cumin cake; CuS2: 2% of cumin seed powder; CuS4: 4% of cumin seed powder; CuS6: 6% of cumin seed powder; CarC2: 2% of caraway cake; CarC4: 4% of caraway cake; CarC6: 6% of caraway cake; CarS2: 2% of caraway seed powder; CarS4: 4% of caraway seed powder; CarS6: 6% of caraway seed powder.

To determine the influence of cumin and caraway powder seeds and by-products on protein bread quality and chemical composition, cumin seeds and by-products were added at 2%, 4% and 6% of whole wheat flour amount, while caraway powder seeds and by-products were added at 2%, 4% and 6% of whole wheat flour amount. A control bread (C) was used for comparison where non seeds or by-products were added to the mixture. All ingredients were mixed for 5 ± 1 min using a dough mixer BEAR Varimixer AR10 (Wodschow & Co., Brondby, Denmark). Dough samples were fermented for 25 min at 36 ± 2 °C temperature. Bread samples were then baked at 200 ± 5 °C temperature for 20 min in a rotating connection oven (Sveba Dahlen, Sweeden) and then cooled at room temperature 22 ± 2 °C for 2 h (Figure 1). 

### 2.4. Sensory Evaluation of Protein Bread

Hedonic scale was used to measure food preferences. Bread samples were analyzed by 60 panelists of both sexes aged 18–46 years students’ and staff at the Faculty of Food Technology, Latvia University of Agriculture. Sensory tests were carried out in a sensory evaluation room in the university, with white light, controlled ventilation, and away from distractions noise, odors and the preparation. Of the 60 participants, 30.2% were male and 69.8% female, 89.4% were aged between 18 and 26 years, and 10.6% from 27 to 46 years. The samples were presented to the participants in identical containers labelled with randomized 3-digit numbers. The samples were presented to the participants in the shape of small squares, they were put in identical containers labelled with randomized 3-digit code. Bread fortified with cumin and caraway flour were analyzed separately. Two glasses of water and green tea had been given to each student in order to overcome the carry-over effects. An acceptance test was applied to attribute the degree of preference using a 5-point hedonic scale (5 = like extremely; 3 = neither like nor dislike; 1 = dislike extremely).

### 2.5. Protein Bread Moisture Content 

The moisture content of protein bread is determined by the mass loss of 1 g of bread sample which has been oven-dried at 103 °C until a constant mass is obtained. Measurements were made in triplicate. 

### 2.6. Protein Bread Crumb Hardness 

Protein bread hardness test was performed on the day of baking, at least 2 h after baking. The hardness of experimental bread samples was measured using TA-XT plus Texture Analyzer (Stable Micro Systems Ltd., Surrey, UK) with the following parameters: probe—a 25 mm diameter aluminium cylinder; test speed —1 mm∙s^−1^; trigger force—0.049 N and distance—4 mm to the bread slice. All values are given as average of six measurements. 

### 2.7. Protein Bread Crumb Color 

To measure the color of bread samples, a Color Tec-PCM/PSM (Accuracy Microsensors Inc., Pittsford, New York, USA) was used based on CIE L*a*b* colour system. In CIE L*a*b* colour system: for L*, 0 = black, 100 = white; for a*, +value = red, −value = green; for b*, +value = yellow, −value = blue. Color was measured at five different points within crumb region; mean values were reported for each sample. 

The total color difference (Δ*E*) was defined by the Minolta Equations (1&2):

∆𝐿 = (𝐿 − 𝐿_0_); ∆𝑎 = (𝑎 − 𝑎_0_); ∆𝑏 = (𝑏 − 𝑏_0_) (1)

(2) $$\Delta E=\sqrt{\Delta L^{2}+\Delta a^{2}+\Delta b^{2}}$$

where: *L*, *a* and *b*—measured values of protein bread samples with cumin or caraway flour; *L*_0_, *a*_0_ and *b*_0_—the values of the protein bread (control). 

### 2.8. Extraction and Determination of Phenolic Compounds from Protein Bread 

One g of protein bread was extracted with ethanol / acetone / water (*v*/*v*/*v* = 7/7/6) solution in an ultrasonic bath WiseClean (GmbH witeg Labortechnik, Wertheim, Germany) at 35 kHz for 10 min at 20 ± 1 °C [14]. Then, the mixture was centrifuged in a centrifuge CM-6MT (Elmi Ltd., Riga, Latvia) at 3500 rpm for 5 min. Thereafter residual bread was re-extracted with the same procedure and supernatant was combined. Triplicate extraction process was done for each sample. 

Total phenolic content (TPC) of the protein bread extract was determined by Folin-Ciocalteu method [15] with some modifications. 0.5 mL of extract was mixed with 2.5 mL of Folin–Ciocalteu reagent (diluted 10 times with water), 3 min later, 2 mL of sodium carbonate (Na_2_CO_3_) (75 g∙L^−1^) was added and mixed. The mixture was placed in the dark at room temperature for further 30 min, and absorbance was measured at 765 nm. TPC values were calculated from the calibration curve of Gallic acid, and the results were expressed as Gallic acid equivalents (GAE) 100 g^−1^ dry weight (DW) of the samples. Measurements were made in triplicate for each extract.

### 2.9. Determination of Trolox Equivalent Antioxidant Capacity (TEAC)

Antioxidant activity of extracts was measured with the 2,2-diphenyl-1-picrylhydrazyl (DPPH) method [16] with slight modifications. A solution of DPPH was freshly prepared by dissolving 4 mg DPPH in 100 mL methanol. Half a milliliter of extract was added into a sample cavity containing 3.5 mL of DPPH solution. Then the mixture was incubated in the dark for 30 min at room temperature. The absorbance was measured at 517 nm using a UV-VIS (ultraviolet-visible) spectrophotometer JENWAY 6300 (Barloworld Scientific Ltd., Staffordshire, UK). The radical scavenging activity was expressed as Trolox mM equivalents (TE) 100 g^−1^ dry weight (DW) of the samples. Measurements were made in triplicate for each extract.

### 2.10. Theoretical Calculation of Protein Bread Nutritional Value 

Nutritional value of protein bread was calculated using conversion factors according to EU Regulation No 1169/2011 [17] on the provision of food information to consumers:

* Carbohydrates (except polyols): 4 kcal∙g^−1^;
* Protein, 4 kcal∙g^−1^;
* Fat, 9 kcal∙g^−1^;
* Fibre, 2 kcal∙g^−1^.

### 2.11. Statistical Analyses 

All experiments were performed in triplicate and the results were presented as the mean ± SD (standard deviation). The values were reported as mean. One-way ANOVA and Tukey test by pairwise at 5% probability level were used for the analyses. 

## 3. Results and Discussion

### 3.1. Protein Bread Sensory Analysis

**Figure 2:**
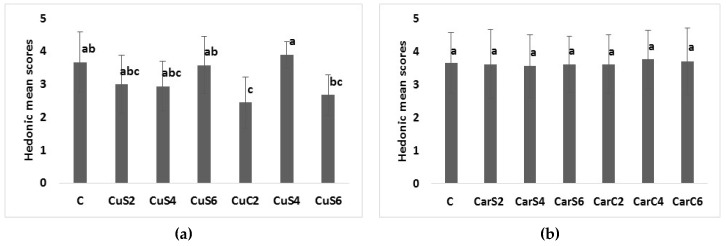
Mean values for overall acceptance of protein bread samples fortified with (**a**) cumin and (**b**) caraway seeds and by-products. CuC2: 2% of cumin cake; CuC4:4% of cumin cake; CuC6: 6% of cumin cake; CuS2: 2% of cumin seed powder; CuS4: 4% of cumin seed powder; CuS6: 6% of cumin seed powder; CarC2: 2% of caraway cake; CarC4: 4% of caraway cake; CarC6: 6% of caraway cake; CarS2: 2% of caraway seed powder; CarS4: 4% of caraway seed powder; CarS6: 6% of caraway seed powder; Columns marked with the same subscript letters in each bar chart are not significantly different (*p* > 0.05).

Figure 2 shows the mean scores assigned to each sample containing different levels of cumin or caraway substitutions in comparison to the control. 

Significant difference was observed in the overall acceptability of the protein bread samples fortified with cumin seeds and by-products (Figure 2a). Our results showed that the scores generally decreased with increase in cumin seeds substitution when compared to control protein bread. Samples CuS4 and CuS6 had the lowest scores since they had a bitter aftertaste, as reported by several participants. Increased scores were observed with increase in cumin by-products substitution, sample CuC6 was the highest suggesting that the panel preferred the sweet taste and aroma of cumin over the control protein bread.

No significant difference among samples fortified with caraway powder seeds and by-products (Figure 2b). Yet, they were all accepted given that all scores were higher than three due to the fact that Latvians are familiarized with bread mostly spiced with caraway seeds. Several participants did not find an impact of bread fortification with cumin and caraway flour on the overall acceptability of protein bread since they did not have a strong influence on the final bread taste and aroma. 

Our overall results revealed that protein bread fortified with by-products flour showed more acceptability than both control bread and bread fortified with seeds flour as they improve the sensory properties of the samples without affecting bread aftertaste.

### 3.2. Protein Bread Color Analysis

Colour is the first feature that consumers rely on for any food product’s acceptance. Mean protein bread colour values with different levels of substitution of cumin and caraway flour along with control bread are presented in Table 1. Results showed that seeds and by-products flour addition led to significantly lower luminosity values of protein bread samples, while redness and yellowness parameters were significantly higher compared to control protein bread.

Increasing the levels from 0 to 6% of cumin seeds and by-products led to a 16 % and 7.75% of reduction in lightness (L*), respectively; a* values increased more than 11% in CuS6 and 6% in CuC6 compared to control bread. The values of b* values also increased about 11% in CuS6 and CuC6 samples compared to control bread. Similar trend was observed in the case of addition of caraway seeds and by-products flour (Table 1). Overall results showed that the increase of substitution levels is accompanied with increase of L* values and decrease of a* and b* values which indicate that browner bread were obtained.

Total color difference (Δ*E*) is a combination of L*, a* and b* values generally used to illustrate bread color variation. Δ*E* values revealed that incorporation of cumin and caraway flour resulted in high color changing (Table 1).

Our findings are in line with those of Tarek-Tilistyak et al. (2015) where darker bread was obtained after addition of linseed oil-seed pressing residues [11]. Besides, darker bread colour was obtained in samples fortified with cumin and caraway by-products flour than bread fortified with seeds flour. The results showed also that bread samples fortified with caraway flour were browner than those fortified with cumin flour (Table 1). Colour changing can be attributed to Maillard reaction which makes browning reaction between amino acids and sugars and to the differences in moisture content between bread samples which also influence the Maillard reaction. The brown colour of added cumin and caraway flour also had a great impact on the final colour of bread samples resulting with darker protein bread [18].

### 3.3. Protein Bread Moisture Content Analysis 

**Figure 3:**
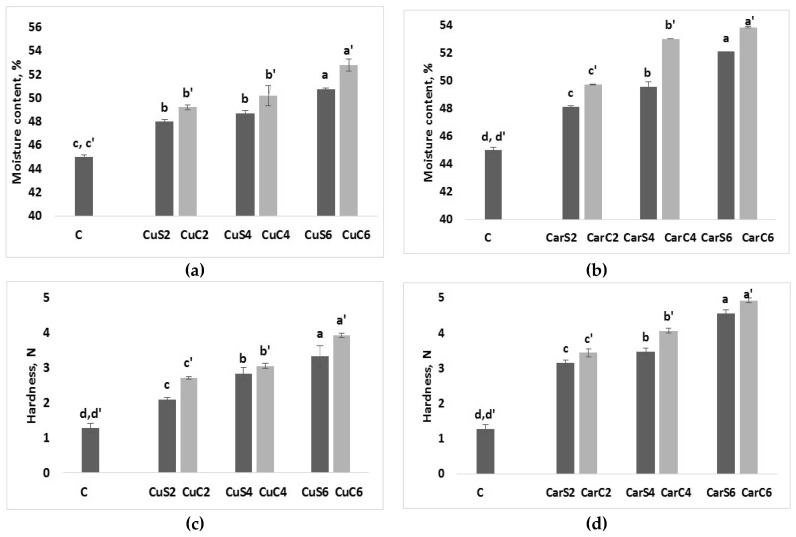
Moisture content (%) and hardness (N) of protein bread fortified with cumin (**a** and **c**) and caraway (**b** and **d**) seeds and by-products. CuC2: 2% of cumin cake; CuC4:4% of cumin cake; CuC6: 6% of cumin cake; CuS2: 2% of cumin seed powder; CuS4: 4% of cumin seed powder; CuS6: 6% of cumin seed powder; CarC2: 2% of caraway cake; CarC4: 4% of caraway cake; CarC6: 6% of caraway cake; CarS2: 2% of caraway seed powder; CarS4: 4% of caraway seed powder; CarS6: 6% of caraway seed powder; Columns marked with the same subscript letters in each bar chart are not significantly different (*p* > 0.05).

Moisture content is a key parameter used to determine bread shelf-stability and susceptibility to microbial infections. The proximate moisture content of protein bread fortified with cumin and caraway powder seeds and by-products are shown in Figure 3. A significant increase of moisture content was obtained in fortified bread samples comparing to control bread.

The moisture content of protein bread increased nearly 6% and 8% in samples fortified with cumin seeds and by-products flour compared to control bread, respectively (Figure 3), and also about 8% and 10% in bread fortified with caraway seeds and by-products flour compared to control bread, respectively (Figure 3).

The overall analysis of protein bread samples revealed that addition of cumin and caraway seeds and by-products flour led to a significant increase of crumb moisture content, this can be attributed to the higher crumb moisture retention caused by the introduction of cumin and caraway powder. A similar trend was obtained by Bansal et al. (2015) who studied the effect of bread fortification with soya flour blends [19]. Furthermore, moisture content of protein bread fortified with by-products flour was higher than those fortified with seeds flour which can be due to the substantial amount of protein and fiber contents as a result of the defatting process. In addition, protein bread with added caraway powder has higher moisture content than bread with added cumin flour. This increase in water retention was most likely due to the higher fiber content in bread fortified with caraway flour resulting by a higher water holding capacity [20].

### 3.4. Protein Bread Hardness Analysis

Figure 3 lists the hardness profile of analyzed protein bread samples. The hardness of protein bread crumbs was positively related to the level of fortification and a significant hardness increase was observed. Crumb hardness increased more than two times in bread fortified with cumin flour (CuS6 and CuC6), and more than three times in bread fortified with caraway flour (CarS6 and CarC6) compared to control bread (C). These results are in agreement with the work of Das et al. (2013) who studied the effect of fennel fortification on the bread firmness [21].

However, hardness profile of protein bread fortified with by-products was higher than bread fortified with seeds flour. Hardness increase might be due to the higher fiber content which is generally accompanied with restriction of gas cells expansion, resulting by a compact structure of bread [22]. Moreover, since the plasticizing effect of water in the bread, hardness increase is also attributed to the increase of moisture content in protein bread samples [23].

### 3.5. Nutritional Values of Protein Bread

Calculated nutrient content and energy values of protein bread samples enriched with cumin and caraway seed and by-product are given in Table 1. Generally, as the level of fortification increased in the all formulations carbohydrate, protein, fiber and fat content increased in comparison with control bread, this increasing amount of nutrients is responsible for the observed increasing energy values in all fortified bread samples compared to control bread (Table 2). However, carbohydrate, protein and fiber content were higher in bread samples fortified with by-products flour than those fortified with seeds flour while fat content was the highest in bread fortified with seeds flour due to the lower fat content in initial by-products flour in both cases. This latter fact was expected as the seeds powder contains more lipids while by-products resulted from defatted seed. These results are in line with previous investigation on the effect of the addition of fully fat and defatted flaxseed flour on wheat bread [24].

### 3.6. Total Phenolic Content (TPC) Analysis

Phenolic compounds are plant secondary metabolites which act as antioxidants owing to their redox properties, consumption of food with high phenol content is highly recommended due to their health promoting effects as they are involved in the prevention of many diseases such as cancers, diabetes and cardiovascular diseases [25].

**Figure 4:**
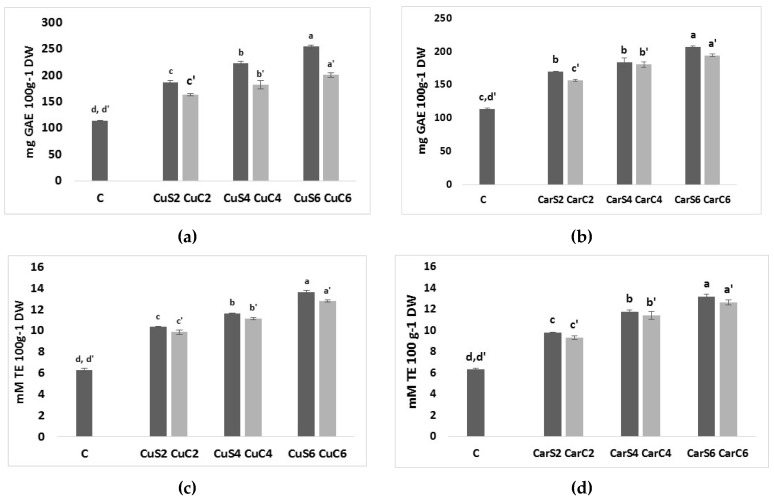
Total phenolic content (expressed as mg Gallic Acid Equivalent (GAE) 100 g^−1^ DW (dry weight)), Trolox equivalent (TE) antioxidant capacity (TE expressed as mM TE 100 g^−1^ DW) of protein bread fortified with cumin (**a** and **c**) and caraway (**b** and **d**) seeds and by-products. CuC2: 2% of cumin cake; CuC4:4% of cumin cake; CuC6: 6% of cumin cake; CuS2: 2% of cumin seed powder; CuS4: 4% of cumin seed powder; CuS6: 6% of cumin seed powder; CarC2: 2% of caraway cake; CarC4: 4% of caraway cake; CarC6: 6% of caraway cake; CarS2: 2% of caraway seed powder; CarS4: 4% of caraway seed powder; CarS6: 6% of caraway seed powder; Columns marked with the same subscript letters in each bar chart are not significantly different (*p* > 0.05).

The total phenolic content (TPC) of different protein bread fortified with cumin and caraway seeds and by-products are presented in Figure 4. Fortified bread samples had significantly higher TPC than the control protein bread. The TPC of bread fortified, regardless of the added flour, were higher than the TPC of control bread more than two times (Figure 4), This increase in TPC in all cases can be attributed to the high content of phenol in added cumin and caraway flour which agrees with previous studies such as the addition of sweet-lupines and rice bran [26,27]. However, bread samples fortified with cumin flour showed greater phenolic content than those fortified with caraway flour which could be attributed to the highest phenolic content in cumin seed [3]. The TPC of bread fortified with by-products flour was lower than the TPC of bread fortified with seeds flour due to the process of defatting which is responsible of the loss of some lipophilic phenolic compounds [16].

### 3.7. Trolox Equivalent Antioxidant Capacity (TEAC) Analysis

Trolox equivalent antioxidant capacity (TEAC) assay is a rapid, simple and inexpensive method employed for determining antioxidant capacity, it measures the ability of a compound to act as free radical or hydrogen donor, and thus it is widely used to evaluate antioxidant activity of food for both lipophilic and hydrophobic antioxidants [28]. The total antioxidant activities (TEAC) of bread fortified with cumin and caraway seeds and by-products flour are shown in Figure 4. TEAC values were strictly dependent on the level of fortification and the differences between control bread and fortified bread were statistically significant.

TEAC values increased with increasing of fortification level of cumin (CuS6 and CuC6) and caraway (CarS6 and CarC6) flour about two times in comparison with control bread (Figure 4). Higher TEAC values means greater antioxidant activity, nonetheless, our results are in accordance with previous studies that reported the positive effect of bread fortification on its antioxidant properties [21,29].

The correlation coefficients (*R*^2^) of total antioxidant activity (TEAC) and total phenolic content (TPC) of the protein bread fortified with seeds and by-products flour were 0.98 and 0.99 in both cases, respectively, which is in line with several previous studies [30,31].

## 4. Conclusion

This study showed the positive impact of bread fortification with different levels of cumin and caraway seeds and by-products fortification on the protein bread quality and overall acceptance. Regarding the organoleptic properties, the percentage should not exceed 4% for cumin and caraway seeds flour and 6% for cumin and caraway by-products flour, respectively. This fortification was advantageous due to the increased nutritional value and higher moisture content with acceptable rheological and sensory features. However, daily intake of fibers and oils containing monounsaturated fatty acids provides many health benefits such as improvement of cardiovascular health and the digestion system. It could also be concluded that bread production may be an ideal alternative for the valorization of cumin and caraway residual by-products.

## Figures and Tables

**Table 1:** Abbreviations of the samples used in the present article, crumb color analysis and total colour difference (Δ*E*) values of protein bread fortified with cumin and caraway seeds and by-product.

Bread Samples	Abbreviations	L*	a*	b*	Δ*E* Values
C	Control	61.08 ± 2.06 ^a^	0.47 ± 0.69 ^d^	20.32 ± 1.96 ^c^	-
CuS2	2% of cumin powder seed	55.79 ± 0.52 ^cd^	1.21 ± 0.04 ^cd^	20.56 ± 0.01 ^c^	5.35
CuS4	4% of cumin powder seed	53.77 ± 0.75 ^d^	3.59 ± 0.03 ^b^	21.19 ± 0.09 ^bc^	8.01
CuS6	6% of cumin powder seed	50.86 ± 0.89 ^e^	5.48 ± 0.65 ^a^	22.56 ± 0.46 ^ab^	11.62
CuC2	2% of cumin by-product	58.90 ± 1.18 ^ab^	0.90 ± 0.01 ^cd^	20.16 ± 0.79 ^c^	2.22
CuC4	4% of cumin by-product	57.69 ± 0.14 ^bc^	1.70 ± 0.39 ^c^	22.44 ± 0.55 ^ab^	4.18
CuC6	6% of cumin by-product	56.35 ± 0.12 ^bcd^	3.09 ± 0.05 ^b^	23.59 ± 0.46 ^a^	6.13
CarS2	2% of caraway powder seed	58.21 ± 0.07 ^b^	1.32 ± 0.11 ^d^	20.37 ± 0.43 ^d^	2.04
CarS4	4% of caraway powder seed	57.72 ± 0.27 ^b^	3.20 ± 0.09 ^b^	22.66 ± 0.82 ^c^	3.84
CarS6	6% of caraway powder seed	56.34 ± 0.30 ^b^	4.94 ± 0.77 ^a^	26.98 ± 1.03 ^a^	9.31
CarC2	2% of caraway by-product	59.03 ± 0.13 ^ab^	1.28 ±0.31 ^d^	22.43 ±0.24 ^bc^	3.05
CarC4	4% of caraway by-product	58.33 ± 0.81 ^b^	2.19 ±0.08 ^c^	24.09 ±0.54 ^bc^	4.97
CarC6	6% of caraway by-product	57.70 ± 0.38 ^b^	3.49 ±0.04 ^b^	25.83±0.58 ^ab^	7.13

* Values marked with the same subscript letters in columns are not significantly different (*p* > 0.05).

**Table 2:** Calculated nutritional and energy values of whole wheat, cumin and caraway seeds and of protein bread fortified with cumin and caraway seeds and by-products.

Bread Samples	Nutrients (g∙100 g^−1^)				Energy Value (kcal∙100 g^−1^)
	Carbohydrates	Protein	Fiber	Fat	
Whole wheat	59.70	11.90	11.20	2.30	340
Cumin seed	44.24	17.81	10.50	22.27	375
Caraway seed	49.90	19.77	38.00	14.59	333
C	25.59	22.37	4.96	0.97	210.49
CuS2	25.77	22.4	5.01	1.2	213.50
CuS4	25.95	22.42	5.06	1.42	216.38
CuS6	26.13	22.45	5.11	1.65	219.39
CuC2	25.93	22.48	5.05	1.09	213.55
CuC4	26.27	22.58	5.14	1.22	216.66
CuC6	26.60	22.69	5.22	1.34	219.66
CarS2	25.82	22.42	5.24	1.14	213.70
CarS4	26.04	22.47	5.51	1.31	216.85
CarS6	26.26	22.52	5.78	1.48	220.00
CarC2	26.00	22.51	5.38	1.06	214.34
CarC4	26.41	22.65	5.78	1.15	218.15
CarC6	26.81	22.79	6.19	1.24	221.94
